# Rare case of acute transverse myelitis associated with Lyme neuroborreliosis

**DOI:** 10.1016/j.idcr.2022.e01422

**Published:** 2022-01-24

**Authors:** Mujtaba Chohan, Dhara Rana, Nagwa Hafez

**Affiliations:** a Rowan University School of Osteopathic Medicine, Stratford, NJ, USA; b Department of Internal Medicine, St. Joseph’s University Medical Center, Paterson, NJ, USA

**Keywords:** Lyme disease, Neuroborreliosis, Acute transverse myelitis, Peripheral neuropathy, Ceftriaxone

## Abstract

Acute transverse myelitis is a neurological disorder that leads to acute spinal cord injury due to inflammation caused by autoimmune disorders or by parainfectious etiologies. Among the many different infectious causes of transverse myelitis, one of the rare ones is Lyme disease. Here we describe a case of a 62 year old male who presents with bilateral paresthesia and weakness. MRI imaging of the cervical and thoracic spine led to the initial diagnosis of cervical cord edema leading to the symptoms associated transverse myelitis. However further workup of different infectious causes lead to positive Lyme titers with positive confirmatory ELISA testing. Initiation of Lyme disease treatment with IV ceftriaxone led to the gradual resolution of the symptoms.

## Introduction

Transverse myelitis (TM) refers to the inflammation of the spinal cord. Acute transverse myelitis (ATM) includes cases of TM that develop over minutes, hours, days or even weeks, whereas chronic etiologies of TM develop progressive myelopathies over many months or years [1]. Moreover, ATM is a neurological disorder causing acute spinal cord injury because of inflammation associated with autoimmune disease like multiple sclerosis or parainfectious etiologies such as Lyme disease (LD), CMV or EBV. ATM is a rare neurological syndrome, which has an incidence between 1.34 and 4.6 per million per year with bimodal peaks between ages 10–19 and 30–39 [2]. ATM can be an acute or slow subacute process where symptoms develop several days to weeks. Presentations of ATM typically include bilateral weakness and sensory disturbance below the level of the lesion, sensory alteration, and autonomic dysfunction such as bowel and bladder dysfunction [2], [3]. Diagnosis of ATM is based on clinical symptoms, cerebral spinal fluid (CSF), and spinal neuroimaging. Although there are limited randomized double blind control treatment trials specifically focusing on ATM, numerous case reports and case series have reported patients benefiting from IV corticosteroid and plasmapheresis [1]. If ATM is due to parainfectious etiology, the patient benefits from treating the underlying cause. After the initiation of appropriate therapy, motor impairment from transverse myelitis improves and perceived improvement in sensory symptoms is often variable [1]. We present a patient who developed signs and symptoms of a rare cause of ATM.

## Case presentation

Here we present a 62-year-old male who came to the ED in mid-summer with a chief complaint of bilateral upper extremity paresthesias and weakness, and lack of coordination in the right arm for the past two days. In the history, the patient noted that he has frequent exposure to deers since he literally has the woods in his backyard. The patient did not mention any remarkable hobbies or other exposure in the history. Symptoms started gradually and progressed. The patient denied any radiating pain, any trauma or eliciting factors that could have led to the symptoms. The patient did report intense headaches two weeks ago that resolved by itself within a few days. Other than these symptoms, the patient denied flu-like symptoms and any bowel and bladder dysfunction. On physical examination, the patient had decreased range of motion in the upper extremities bilaterally, lack of coordination in the right arm, and muscle strength of 2/5 in the left arm. Muscle strength in the right upper extremity was 3/5 grip strength, 5/5 elbow flexion, 4/5 wrist flexion, 2/5 wrist extension, 4/5 shoulder abduction. Muscle strength of the left upper extremity showed 3/5 grip strength, 1/5 elbow flexion, 5/5 wrist flexion, 4/5 wrist extension, 1/5 shoulder abduction. Muscle strength of the lower extremities was 5/5 bilaterally. Sensory function was intact bilaterally in both upper and lower extremities.

Vitals and blood work were unremarkable except neutrophil percent of 91.4 with a normal WBC (5.5 × 10^3^/mm^3^). MRI was consistent with nonspecific transverse myelitis and CT scan was positive for multilevel degenerative disc disease and facet osteoarthritis.

Initial suspicion was toward cervical edema, but further workup was done to rule out any mass lesions of the cervical spine, inflammatory diseases, and infectious etiologies. The patient had positive Lyme serum titers (Lyme IgG 1.38 and Lyme IgM 2.71). Serum PCR testing for other infections (i.e. influenza A & B, HSV, CMV, Coronavirus, Rhinovirus, Enterovirus, Parainfluenza virus, RSV, B. pertussis, C. pneumoniae, M. pneumoniae, E. coli, H. influenza, N. meningitis, S. agalactiae, S. pneumoniae, HSV 1 & 2 and West Nile Virus) were negative. Serum testing for connective tissue antibodies were also negative (i.e. Scl-70, Smith, SSA, SSB).

Lumbar puncture was performed and was indicative for meningitis. CSF values consisted of WBC of 720 mm^3^, RBC of 2 mm^3^, protein of 168 mg/dL, glucose of 67 mg/dL, in addition to positive Lyme titers. CSF WBC was lymphocytic predominant with normal segmented neutrophils (1%), elevated lymphocytes (89%), and low monocytes (10%). The CSF western blot showed positive IgG (present for P18, P23, P39, P41, P45, and absent for P26, P30, P58, P66, P93) and IgM for Borrelia-specific bands (present for P23,39,41). The CSF PCR for HSV 1 & 2 were not detected. VDLR was also not detected in the CSF. This confirmed neuroborreliosis-related transverse myelitis. IV Ceftriaxone(2 g) was initiated for a total of 28 days. Two days after the initiation of ceftriaxone, the patient’s muscle strength increased to 4/5 in the right UE and 2/5 in the LUE. A total of four days after the initiation of antibiotics, the patient was able to regain 5/5 muscle strength in both upper extremities throughout. Patient was discharged to acute rehab to get further physical therapy for the upper extremities and received ceftriaxone for a total of 28 days.

### Radiology

**Fig. 1:**
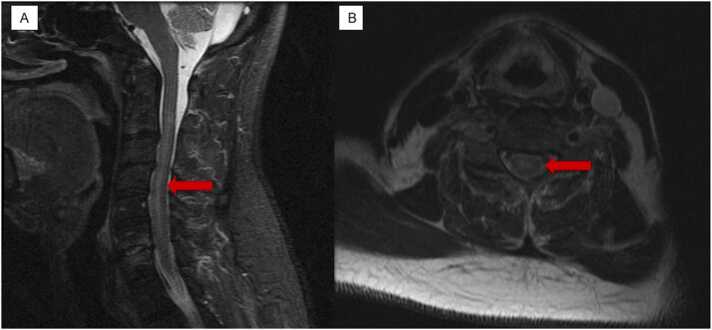
**A**. STIR sagittal cervical spine MRI with and without contrast. The red arrow shows edema within central cord. **B**. T2 Axial cervical spine MRI with and without contrast showing subtle hyperintensity without the spinal cord indicating spinal cord edema.

The initial cervical MRI without contrast showed cervical level C4-C5 moderate canal stenosis and subtle T2 hyperintense signal within the cord, which may represent cord edema (Fig. 1A, B). Additionally, on the cervical level C5-C6 there is broad-based disc bulge with moderate canal stenosis and severe left neural foraminal narrowing. There is a suspected T2 hyperintense signal at the level of the cord. The final impression demonstrated severe discogenic degenerative disease that is most notable at C4-C5 and C5-C6 where there is moderate canal stenosis and possible cord edema versus myelomalacia.

## Discussion

Lyme disease is a multistage and multi-organ infectious disease caused by the spirochete *borrelia burgdorferi*, which is transmitted to humans from a bite of the tick species, *Ixodes scapularis* and *Ixodes pacificus* [4]. After the transmission of the spirochete from the tick bite, different clinical manifestations of the disease occur in stages. Neurological manifestations from LD are called neuroborreliosis. There are two distinct types of neuroborreliosis: second stage Lyme meningitis and third stage brain parenchymal involvement. The second stage Lyme meningitis resembles aseptic meningitis with facial palsies, cranial neuritis, peripheral nerve involvement, and radiculopathies [5], [6], [7]. The third-stage Lyme neuroborreliosis involves the brain parenchyma that causes a multitude of nonspecific CNS manifestations that can be confused with multiple sclerosis, brain tumors, and psychiatric derangement [6], [7].

Our patient presented with typical symptoms and signs of ATM with gradual worsening of bilateral upper extremity weakness and acute onset of severe headache that started two weeks prior to the ED visit. Furthermore, the patient had no presence of classic “bull’s eye rash” but had neurological symptoms on physical exam. The patient’s MRI indicated spinal cord compression due to edema, positive serology for Lyme and positive CSF western blot for Lyme. Though at initial assessment the parainfectious cause of the neurological symptom may be dismissed, it is vital to keep infectious diseases such as Lyme as potential causes especially of a patient living in an endemic area for Lyme disease.

Lyme neuroborreliosis can be the first evidence of Lyme disease occurring without a history of erythema migran or flu-like illness [7]. A retrospective case series study of 165 patients presenting for possible early Lyme disease found that of those diagnosed with early Lyme disease 13% did not present with an erythema migran, of those presenting without a rash 54% were misdiagnosed and among those with a rash the diagnosis of erythema migrans was initially missed in 23% of the patients [8].

The aspect that makes our case unique is the presence of disease associated with ATM without the presence of the erythema migran, flu-like symptoms, or radiculopathy. Furthermore, the patient did not exhibit any late occurring symptoms of Lyme disease such as bell’s palsy, heart block, or encephalopathy. ATM account for 4–5% of all cases of neuroborreliosis [9]. Disease associated with ATM can be grouped into three categories: systemic inflammatory disease, infectious disease, and multifocal CNS disease. Infectious myelitis account for about 12% of patients and can be due to viral, bacterial, fungal, or parasitic infections [10]. To confirm suspected cases of ATM, the initial preferred imaging method is brain and spine MRI with or without contrast and a positive study will show cord edema and stenosis [1], [11]. This should be followed up with a lumbar puncture to analyze the CSF for inflammatory markers, oligoclonal bands, and a western blot of the CSF.

Our patient’s cervical spine MRI showed moderate canal stenosis and possible cord edema most notable at the C4-C5 and C5-C6 levels (Fig. 1). Moreover, the patient’s serology indicated high Lyme IgG and IgM levels and the CSF western blot showed positive for Borrelia-specific bands for IgG and IgM. Therefore, making a high suspicion of ATM due to neuroborreliosis. This imaging study and lab findings are consistent with other neuroborreliosis-associated ATM reported in literature where Lyme serology was positive in CSF and T2 weighted-cervical MRI showed spinal cord due to edema [9], [10], [12], [13], [14], [15].

Although there are definitive diagnostic criteria for non-neuroinvasive Lyme disease, currently in the United States the diagnostic testing guidelines for confirmation of Lyme neuroborreliosis are not as well defined. To make a diagnosis of neuroborreliosis, an immunoblot in CSF is often performed to detect IgM and/or IgG class antibodies to *Borrelia burgdorferi* [16]. However, there are no specific criteria established for the interpretation of banding patterns in CSF like the one used to interpret banding patterns in serology [16], [17]. In addition to the CSF immunoblot, the Lyme disease antibody index is used to establish the diagnosis of neuroborreliosis [16], [17]. However, the use of this index for the presence of Borrelia-specific IgM and IgG is not definitive evidence of CSF antibody synthesis due to either peripheral blood contamination from the lumbar puncture or diffusion of serum antibodies in the blood-brain-barrier [16]. In addition, the diagnostic sensitivity of Lyme disease antibody index is largely variable (range 55–100%) due to the duration of symptoms prior to specimen collection [16]. Our lab did not analyze the CSF Lyme disease antibody index because only a limited number of reference laboratories in the United States offer a Lyme disease antibody index [16].

As for the treatment of ATM due Lyme, typically 2–4 weeks of treatment with IV ceftriaxone, penicillin or orally administered doxycycline are recommended [4], [18]. Although most outcomes are favorable, failure and long-term disability may occur [18]. After 4 days of high dose IV ceftriaxone, our patient began to show improvement of his upper extremity weakness on the physical exam.

## Conclusion

ATM is a rare neurological disorder that presents with bilateral muscle weakness and sensory deficits and can possibly affect bowel and bladder function. It is associated with many autoimmune and infectious etiologies. ATM due to infectious etiology itself is a rare cause. Furthermore, neuroborreliosis is also a rare cause of an infectious etiology leading to ATM. Making a diagnosis of ATM due to Lyme can be difficult since the clinical feature of Lyme disease can present with or without the typical features such as the erythema migran or flu like symptoms. Appropriate screening of Lyme disease is warranted in an endemic area. Furthermore, the initiation of treatment can help reverse the neurological deficit that comes with ATM due to neuroborreliosis.

## Funding

This research did not receive any specific grant from funding agencies in the public, commercial, or not-for-profit sectors.

## Ethical approval

No ethical approval was required for this study.

## CRediT authorship contribution statement

**DR** and **MC** contributed equally to this work in study design, data collection, interpretation of data, drafting and revising the article. **HN** supervised this study, patient’s treatment and provided final approval of this version for submission.

## Conflict of Interest Statement

None.
